# Coronary arteriovenous fistulas in the adults: natural history and management strategies

**DOI:** 10.1186/1749-8090-4-62

**Published:** 2009-11-06

**Authors:** Yusuf Ata, Tamer Turk, Murat Bicer, Mihriban Yalcin, Filiz Ata, Senol Yavuz

**Affiliations:** 1 Bursa Yuksek Ihtisas Education and Research Hospital, Department of Cardiovascular Surgery, Bursa, Turkey; 2 Uludag University Medical Faculty, Department of Cardiovascular Surgery, Bursa, Turkey; 3 Bursa Yuksek Ihtisas Education and Research Hospital, Department of Anesthesiology, Bursa, Turkey

## Abstract

**Objective:**

To describe aspects of the natural history and pathophysiology of coronary arteriovenous fistula and to propose potential treatment strategies.

**Methods:**

Eleven adult patients were treated surgically for coronary arteriovenous fistulas (8 male, 3 female) during the last three years. Mean age was 48,7 ± 9,5 years (range 32-65 years). Diagnosis was made by coronary angiography and transesophageal echocardiography

**Results:**

All patients were symptomatic due to the associating cardiac disorder or fistula. Presenting symptoms were chest pain, exertional dyspnea and palpitation. All patients were diagnosed by selective angiography. Transthoracic and transoesophageal echocardiography was performed to identify the Qp/Qs ratio in one patient. One patient who had an LAD to pulmonary artery coronary arteriovenous fistula with a vascular malformation needed early reoperation due to recurrence of the fistula. Echocardiographic evaluation at the postoperative third month revealed no residual shunts in all patients.

**Conclusion:**

Because of the severe complications that may develop due to coronary arteriovenous fistula, we believe that every coronary artery fistula should be treated invasively by surgery or transcatheter closure. But both treatment modalities still need to be evaluated with randomized multicenter studies for long term survival and effectiveness.

## Introduction

Coronary arteriovenous fistula (CAVF) is rare anomaly which consists of abnormal communication between coronary artery and one of the cardiac chambers or vessels adjacent to the heart. Coronary arteriovenous fistulas (CAVFs) are present in 0.002% of the general population and are visualized in nearly 0.25% of patients undergoing catheterization [1-5].

Most of the patients with CAVFs are older than 20 years. Although they remain asymptomatic, symptoms and complications may develop with increasing age, and when surgery is performed in later life mortality and morbidity is increased [6,7]. We present our experience in eleven adult patients with CAVFs, document diagnostic evaluation and management strategies. The objective of this study was to describe aspects of the natural history and pathophysiology of CAVF and to propose potential treatment strategies.

## Methods

### Patients

In the last three years 11 adult patients with CAVFs were treated surgically in two hospitals. The mean age was 48,7 ± 9,5 years (range 32-65 years). Coronary angiography was performed in all patients due to presenting symptoms and associated cardiac disorder. Transthoracic and transoesophageal echocardiography was performed to identify the Qp/Qs ratio in one patient.

All patients were symptomatic, presenting symptoms were angina, exertional dyspnea and palpitation. Clinical symptoms mostly depended on the associated cardiac disorder.

## Results

**Figure 1:**
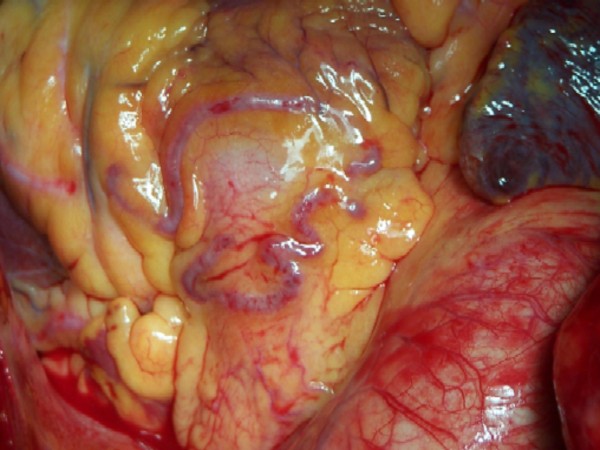
**Coronary arteriovenous fistula between LAD and pulmonary artery**.

**Figure 2:**
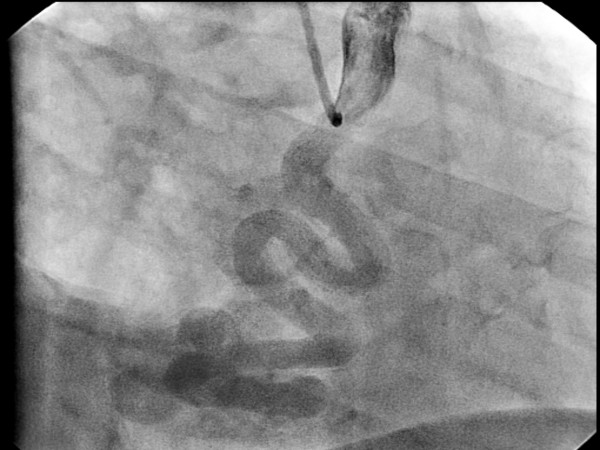
**Dilated right coronary artery and coronary arteriovenous fistula draining into the coronary sinus**.

Coronary angiography revealed 12 CAVFs originating from the proximal left descending artery (n = 8) (Fig 1), the left main coronary (n = 1), and the right coronary artery (n = 3). One patient (9,1%) had bilateral fistulas with origin from the right coronary artery and the left descending artery. The majority of the CAVFs (n = 11) drained into the main pulmonary artery. Only in one case (6.6%) fistula drained from right coronary artery into coronary sinus with an aneurysm of the right coronary artery (Fig 2). RCA to Coronary Sinus fistula patient was evaluated with transthoracic and transosephageal echocardiography which showed normal contractile function with a Qp/Qs ratio 2.4/1.

Surgical correction was performed in all patients via median sternotomy. Seven patients were operated with the use of the cardiopulmonary bypass (CPB). Heparin was administrated (300 U/kg) before aortic cannulation. Anticoagulation was maintained during CPB and monitored with activated clotting time measurements (Hemochron 801, International Technique Corp, Edison, NJ, USA). We performed moderate systemic hypothermia (30°C-32°C) in all the patients. A roller pump, a nonheparinized circuit and a hollow-fiber oxygenator were used. The pump flow was kept between 2.0-2.5 L/min/m2 body surface area to maintain a mean arterial pressure of 50 to 70 mmHg. Ascending aorta was clamped after the clamping of the CAVF near to the drainage area. Myocardial protection was achieved by an initial antegrade infusion of the St. Thomas' crystalloid cardioplegia and then continued with intermittent antegrade cold blood cardioplegia.

Other four patients were operated on beating heart without the use of CPB. After a median sternotomy heparin (150 U/kg) was administered. The Octopus Tissue Stabilizer (Octopus-4, Medtronic, Cardiac Surgical Products, MI, USA) was used for the stabilization of the target coronary artery. Heparin was antagonized with protamin sulphate until the activated clotting time decreased below 200 seconds.

All CAVFs are visible at the surface of the heart and a continuous thrill was palpable over all of the CAVFs. All CAVFs were dissected near their origins and were temporarily occluded with bulldog clamps until the thrill disappeared for 20 minutes. After this period CAVFs were ligated both proximally and distally at the origin and the drainage site. The operations performed in association with closure of the CAVFs are listed in Table 1.

**Table 1:** Origin, drainage site of the CAVFs and the surgical treatment performed

**Patient**	**Age**	**Origin**	**Drainage**	**Treatment**
1	46	LAD	PA	SC+OPCABG
2	50	LAD	PA	SC+OPCABG
3	53	LAD	PA	SC+OPCABG
4	51	RCA	PA	SC+MVR+CABG
5	46	LMCA	PA	SC+CABG
6	56	LAD	PA	SC+CABG
7	58	LAD	PA	SC+CABG
8	37	LAD	PA	SC+OPCABG
9	42	LAD and RCA	PA-PA	SC+CABG
10	32	RCA	CS	SC
11	65	LAD	PA	SC+CABG

LAD, left anterior descending coronary artery; PA, pulmonary artery; SC, surgical closure; OPCABG, off-pump coronary artery bypass grafting; RCA, right coronary artery; MVR, mitral valve replacement; LMCA, left main coronary artery; CABG, on-pump coronary artery bypass grafting

There was no surgical death; only one patient with recurrence of the fistula that was operated on beating heart needed early reoperation. This patient had a vascular malformation located on the main pulmonary trunk, after clamping and surgical ligation of the fistula the thrill disappeared but in the ICU the thrill appeared again. Coronary angiography revealed a fistula between LAD and the pulmonary artery. The patient was reoperated with CPB and the pulmonary connection of the fistula was ligated after opening the pulmonary trunk. All patients underwent echocardiography at the postoperative third month which revealed no residual shunts in all patients.

## Discussion

CAVF is a very rare anomaly. It was firstly described by Krause in 1865 and the first surgical treatment was also done by Bjork and Crafoord in 1947 [8,9]. CAVFs constitute nearly half of all coronary artery anomalies and are the most common of hemodynamically significant coronary lesions [1-7]. Approximately half of all patients with CAVF remain asymptomatic and some CAVF might disappear spontaneously during childhood [4,5,7,10,11].

CAVF may be congenital or acquired. CAVFs are associated with an other congenital heart disease in 20% to 45% and isolated in 55% to 80% of the cases [3,4,6,12]. Associated anomalies include atrial septal defect, tetralogy of Fallot, patent ductus arteriosus, ventricular septal defect, and pulmonary atresia [1,3,4,6].

Origin of the CAVF can be any of the three major coronary arteries, including the left main trunk. The majority of these fistulas arise from the right coronary arteries or the left anterior descending; the circumflex coronary artery is rarely involved [1,4-6]. Single origin is the most common form of CAVF, ranging from 74% to 90% of the cases [1,4,6,12]. The right coronary artery or its branches is the most common site of the CAVFs with 55% and the second common site is the left coronary artery in about 35% of the cases [5]. In contrast with the majority of the literature but similar to the observations of Tirolimis et al. and Carrel et al. most of the CAVFs (75%) in our study group were originating from the left coronary artery and only 3 (25%) CAVFs were originating from the right coronary artery [13,14]. This might be because our small population study group is only consisted of adult patients. Multiple fistulas may be present in 10.7% to 16%, and fistulas might originate from both coronaries in 4% to 18% of the cases [1-6]. One (9%) of our cases also has double CAVFs originating both from right coronary artery and left coronary artery (Table 1).

Over 90% of the fistulas drain into the venous structures of circulation. These include right-sided chambers, pulmonary artery, coronary sinus, and superior vena cava but drainage into the left-sided chambers is less frequent. Fistulous drainage occurs into the right ventricle in 40%, right atrium in 26%, pulmonary artery in 17%, left ventricle in 3%, coronary sinus in 7%, and superior vena cava in 1% [4-6]. Drainage site was into the pulmonary artery in 10 patients and into the coronary sinus in one (Table 1). This difference should be due to our older aged study group which was shown by Urrutia et al. that drainage into the main pulmonary artery are a relatively common occurrence, especially in patients with increasing age [3].

Coronary artery dilatation is common but degree of dilatation does not always depend on the shunt size. In one of our case that was draining into the coronary sinus there was a notable dilatation in the right coronary artery (Fig 2).

The majority of the adult patients remain asymptomatic according to size and localization of the CAVF. Symptomatic adult patients may have symptoms of dyspnea, fatigue, and angina these symptoms might be due to concomitant presence of underlying cardiac disease [3,7,13].

CAVF may result in severe complications; such as pulmonary hypertension because of an existing large left to right shunt, congestive heart failure, subacute bacterial endocarditis, myocardial ischemia resulting from steal phenomena, rupture or thrombosis of the CAVF or associating arterial aneurysm [5,6]. Symptoms and risk of these lethal complications increase with age [6,7,13].

The diagnosis of CAVF challenging as its prevalence is low, yet it should be considered in many symptomatic or asymptomatic patients presenting with cardiac murmurs. Differential diagnosis includes patent ductus arteriosus, pulmonary arteriovenous fistula, ruptured sinus of Valsalva aneurysm, aortopulmonary window, prolapse of the right aortic cusp with a supracristal ventricular septal defect, internal mammary artery to pulmonary artery fistula, and systemic arteriovenous fistula [5,6,15].

Traditional way of diagnosis of CAVF is invasive investigations such as cardiac catheterization and coronary angiography. Most of the fistulas are small and found incidentally during coronary angiography. Coronary angiography still remains the gold standard for imaging the coronary arteries, but sometimes origin and relation of CAVF to adjacent cardiac structures may be ambiguous. It is difficult to measure and observe abnormal tortuous blood vessels with coronary angiography in one section, under such conditions non-invasive methods such as transthoracic echocardiography combined with Doppler and color flow imaging, transoesophageal echocardiography, magnetic resonance imaging and contrast enhanced multislice tomography can be used as adjunct to coronary angiography [5,6,16-18].

Although the natural history of the CAVF is variable and some spontaneous closure is reported it is widely recommended by most of the authors that symptomatic CAVF should be treated, but is still controversial in patients without symptoms [3,7,10,11,19-21]. Some authors recommend closure of CAVF even in asymptomatic patients to prevent fistula related complications those will increase with age, especially because of the risk of heart failure, endocarditis and myocardial ischemia [3,6,13,22]. The authors of the present study accepted this recommendation and proposed closure of the diagnosed CAVFs. Most of the patients presented in our study had concomitant cardiac diseases therefore these diagnosed CAVFs were important in the planning and the performance of the surgical treatment of the coexisting cardiac disease. On the other hand in these cases the closure of the CAVFs should be performed to reduce postoperative early and late complications.

Surgical closure of CAVF by epicardial and endocardial ligations are gold standard for the treatment of CAVF and remains safe and effective with good reported success [13,22-24]. Some authors have reported successful surgical occlusion of CAVF on beating heart without cardiopulmonary bypass [2,25]. Ligation of the CAVF may be performed on the outside of the heart without CPB bypass when there is a simple and easily accessible CAVF. But we recommend exploration of the pulmonary artery with the use of cardiopulmonary bypass especially in patients having a CAVF in combination with a vascular malformation as in our patient that needed reoperation because after the surgical occlusion of the dominant left to right shunt in the CAVF omitted communication in the vascular malformation can cause late recurrence.

There is an increase in TCC treatment of CAVF in recent years with the use of advanced interventional devices [21,26]. TCC closure technique needs several conditions: anatomy of the fistula should be favorable for this treatment (eg. nontortuose vessel, the fistula should be unique with distal narrowing to avoid embolism to the drainage site, and distal portion of the fýstula should be accessible with the closure device [20,21,26].

## Conclusion

In the light of the literature we recommend the following as a treatment strategy: (1) patient with a symptomatic or an asymptomatic CAVF and an additional cardiac pathology that needs surgical intervention should refer to surgical closure; (2) patient with a symptomatic or asymptomatic CAVF and unsuitable anatomy for TCC closure should refer to surgical closure; (3) patient with a symptomatic or an asymptomatic CAVF and a suitable anatomy TCC should refer to TCC; (4) patient with a symptomatic or an asymptomatic CAVF with a coexisting cardiac pathology that needs percutaneous coronary intervention should refer to TCC; and (5) patient with a failed TCC should refer to surgical closure.

In conclusion, surgical closure of the CAVF can be performed with very low risk especially on the beating heart and in cases of isolated CAVF with suitable anatomy TCC is the alternative treatment selection. But yet surgery and especially TCC closure needs to be evaluated with randomized multicenter studies for long-term survival and effectiveness of the both therapeutic modalities.

## Competing interests

Next Pharma financed the article processing charge of this article.

## Authors' contributions

YA participated in collecting the data, writing, reviewing and submitting the manuscript. TT conceived of the study, participated in writing and submitting the manuscript. MB participated in collecting the data and reviewing the manuscript. MY participated in collecting the data and reviewing the manuscript. FA participated in reviewing and in writing of the manuscript. SY participated in reviewing of the manuscript. All authors read and approved the final manuscript.
